# Low resilience as risk factor of mental disorders during COVID-19 pandemic: A cohort study: Pere Castellvi

**DOI:** 10.1093/eurpub/ckac131.477

**Published:** 2022-10-25

**Authors:** P Castellvi, M Llistosella, A Miranda-Mendizabal, S Recoder, E Calbo, M Casajuana-Closas, D Leiva, R Manolov, N Matilla-Santander, C Garcia-Forero

**Affiliations:** 1 Medicine, Universitat Internacional de Catalunya, Sant Cugat del Vallès, Spain; 2 Nursing, Consorci Sanitari de Terrassa, Terrassa, Spain; 3 Nursing, Universitat Internacional de Catalunya, Sant Cugat del Vallès, Spain; 4 Psychology, Universitat Internacional de Catalunya, Sant Cugat del Vallès, Spain; 5 Primary Care, IDIAP Jordi Gol, Barcelona, Spain; 6 Psychology, Universitat de Barcelona, Barcelona, Spain; 7 Occupational Medicine, Karolinska Institutet, Stockholm, Sweden

## Abstract

**Background:**

To analyze whether people with low resilience are at higher risk of mental health problems during the COVID-19 pandemic in Spanish adults.

**Methods:**

A longitudinal cohort study was carried out. Resilience was measured pre/post-pandemic with the CD-RISC. Mental health problems assessed were: Major Depressive Episode (MDE), Generalized Anxiety Disorder (GAD), Suicidal Thoughts and Behaviors (STB), and Posttraumatic Stress Disorder (PTSD) symptoms.

**Results:**

We found statistically significant differences between groups and resilience scores in MDE [F (3;48.40)=19.55], GAD [F(3;19.63)=6.45] and STB [F(3;111.74)=31.94]. Multivariable analyses showed individuals with very low resilience were at a 5-fold risk of Incidence of MDE and a 4-fold risk of STB. Persistent group presented a 21-fold risk of MDE and 54-fold risk of STB, respectively. No evidence of higher risk was found for GAD. Individuals with low resilience and exposed to COVID-19 did not have a significantly higher risk. Individuals with low resilience were at higher risk of PTSD in general population [β(95%CI)= -3.25(-3.969 to -2.54)], but not for individuals with COVID-19.

**Conclusions:**

In the general population, having low or very low resilience increases the risk of suffering MDE, STB, and PTSD, but not GAD during the COVID-19 pandemic, but not in the population with COVID-19.

**Key messages:**

• Resilience was a buffer of mental health problems in general population, but not in those exposed to COVID-19.

• Those with low resilience were at 21-fold risk of mental health problems before and during the first year of pandemic.

